# Biologically Inspired Optimal Terminal Iterative Learning Control for the Swing Phase of Gait in a Hybrid Neuroprosthesis: A Modeling Study

**DOI:** 10.3390/bioengineering9020071

**Published:** 2022-02-12

**Authors:** Nathaniel S. Makowski, Marshaun N. Fitzpatrick, Ronald J. Triolo, Ryan-David Reyes, Roger D. Quinn, Musa Audu

**Affiliations:** 1 Department of Physical Medicine and Rehabilitation, MetroHealth System, Cleveland, OH 44109, USA; 2 APT Center, Louis Stokes VA Medical Center, Cleveland, OH 44106, USA; ronald.triolo@case.edu (R.J.T.); mxa93@case.edu (M.A.); 3 Department of Mechanical and Aerospace Engineering, Case Western Reserve University, Cleveland, OH 44106, USA; mnf13@case.edu (M.N.F.); rdq@case.edu (R.D.Q.); 4 Department of Biomedical Engineering, Case Western Reserve University, Cleveland, OH 44106, USA; rxr410@case.edu

**Keywords:** electrical stimulation, exoskeleton, neuroprosthesis, cooperative control, musculoskeletal model

## Abstract

(1) Background: An iterative learning control (ILC) strategy was developed for a “Muscle First” Motor-Assisted Hybrid Neuroprosthesis (MAHNP). The MAHNP combines a backdrivable exoskeletal brace with neural stimulation technology to enable persons with paraplegia due to spinal cord injury (SCI) to execute ambulatory motions and walk upright. (2) Methods: The ILC strategy was developed to swing the legs in a biologically inspired ballistic fashion. It maximizes muscular recruitment and activates the motorized exoskeletal bracing to assist the motion as needed. The control algorithm was tested using an anatomically realistic three-dimensional musculoskeletal model of the lower leg and pelvis suitably modified to account for exoskeletal inertia. The model was developed and tested with the OpenSim biomechanical modeling suite. (3) Results: Preliminary data demonstrate the efficacy of the controller in swing-leg simulations and its ability to learn to balance muscular and motor contributions to improve performance and accomplish consistent stepping. In particular, the controller took 15 iterations to achieve the desired outcome with 0.3% error.

## 1. Introduction

Restoration of walking is a high priority for persons with paraplegia due to SCI, with one solution being rehabilitative exoskeletons [1]. Commercially available robotic exoskeletal walking assist devices currently on the market include both untethered devices intended for community use and as therapeutic interventions such as Rex, ReWalk, Ekso, and Indego [2] as well as mounted systems that are incapable of overground walking and are only intended as a therapeutic tool, such as the Lokomat [3].

These exoskeletons generate ambulatory motions with mechanical actuators mounted on external bracing worn on the user’s, or pilot’s, body. They place the pilot in an upright position as opposed to sitting in a wheelchair, which generates health benefits such as better bowel and bladder function and spasticity reduction [4]. However, commercial systems are motor driven and do not activate the paralyzed lower extremity muscles to contribute to walking motions, leaving the lower limbs to continue to atrophy.

Hybrid exoskeletons, or hybrid neuroprostheses, combine exoskeletal bracing with neuromuscular stimulation to take advantage of biological motive power, with activation of paralyzed muscles, thereby mitigating atrophy [5,6]. These devices use implanted or surface electrodes to deliver neural stimulation to recruit otherwise paralyzed muscles to produce movement coordinated with active assistance from exoskeletal bracing.

There are a variety of methods to integrate synergistic forces generated by lower extremity muscles with torques generated by motorized bracing to accomplish ambulatory motion, but certain characteristics of this combined system make coordination difficult. Muscle contractions elicited by neuromuscular stimulation represent highly nonlinear time-varying systems, in contrast to the motorized bracing, which can be effectively modeled as linear time-invariant systems [5]. Such hybrid systems also exhibit actuator redundancy, with multiple muscles as well as a motor acting on the same joint, allowing a multiplicity of solutions that can generate the same desired joint torque [7].

One of the simplest methods of coordination is to perform trajectory control with the exoskeleton while activating muscles to reduce control effort and inhibit muscle spasticity during movement [8]. However, this controller does not intelligently allocate control effort between muscle and motor, as the primary aim of the control law is to reduce spasticity.

In the switching control method described in [9], the hybrid neuroprosthesis alternates between two modes to control the knee joints: muscular stimulation only and motorized bracing only. The controller applies a variable-gain proportional derivative (PD) controller with delay compensation to the muscles controlling the knee joint, while internally modelling an estimate of muscle fatigue. Once estimated fatigue exceeds a pre-specified threshold, the hybrid neuroprosthesis switches to motor control. This approach sidesteps the redundant actuation problem by ensuring that only one set of actuators is active at a time.

Several methods have been proposed that utilize internal models to estimate unobservable states of the system and distribute control effort across muscles and motorized bracing. The FEXO Knee, described in [10], controls muscles with a feedforward controller that incorporates an inverse muscle model, while applying a PD controller to the motors to enforce a reference trajectory. An optimization algorithm updates the distribution of torque between knee muscles and knee motors. This algorithm is designed to keep the contribution from neural stimulation at a fixed amount and only updates motor contributions over time. The study described in [11] reports a similar method of control with an inverse muscle model. It implemented an extended optimization for joint torque distribution over multiple redundant agonist muscles and actuators and factored in an internal estimate of muscle fatigue.

The Exoskeleton Intelligently Communicating and Sensitive to Intention (EICOSI) implements a nonlinear disturbance observer for on-line estimation of generated stimulation torque, which varies motor assistance based on this estimation [12]. The control method described in [13] incorporates a model predictive controller to generate a desired overall torque and a second stage which splits the torque contribution between the muscles and motors via a low-pass filter. The muscle performs the low-frequency elements prescribed by the control signal, and the motor performs the high-frequency portions.

There are several studies that augment model predictive control with nonlinear strategies to control hybrid neuroprostheses [14,15,16]. These methods incorporate optimal predictive control to determine the best allocation of control inputs across multiple redundant actuators that act on a single joint and nonlinear techniques that account for complex nonlinear dynamics arising from biological aspects of the system, such as muscle activation dynamics and electromechanical delay. However, all previously described methods that incorporate an internal model require a sufficiently accurate representation of the system for good performance, as well as a system identification phase to generate model parameters unique to each pilot. The method described in [17] implements model predictive control but replaces the internal model with a recurrent neural network. This method is computationally complex and would require training the neural network separately to model the capabilities of each pilot.

Iterative learning control (ILC) provides a simple yet robust method for improving controlled system performance over time. ILC algorithms intelligently learn from errors in the previous iterations to improve performance on the next iteration. This technique was originally developed for robotics [18,19] but has since found diverse application in circuit fabrication [20], transportation [21], and even agriculture [22]. It exploits a system that performs repetitive motions and exhibits repetitive measurable errors.

ILC has been extended to control of multi-joint neural stimulation alone [23], as well as interventions combining neural stimulation and exoskeletal motors. There are several hybrid exoskeleton control methods that treat gait as a repetitive, cyclic system and use ILC-inspired methods to achieve coordination between the activated lower limb muscles and the exoskeleton motors. The simplest augments the Indego commercial exoskeleton with stimulation modulated by an ILC algorithm on subsequent steps based on the control effort of the actuators to track a predefined trajectory [24]. Similarly, the Kinesis implements ILC to iteratively update the torque produced by the muscles with the goal of minimizing the interaction torque between the pilot’s limb and the device [25]. The ILC in these applications does not modulate motor torque, which is the responsibility of a higher-level controller.

In [26], ILC estimates system dynamics to inform a sliding-mode controller. The system then switches between motor or muscle activation to control the joint depending on an estimate of muscle fatigue. This method is extended in [27], where a neural network-based ILC is applied to learn the system dynamics but requires an additional model predictive controller to allocate control effort between the redundant actuators. Major limitations of some of these controller designs include (1) requiring a good model of the system, which is difficult to assemble and changes from user to user; (2) the need for additional system identification, which could be expensive for whole-body systems; and (3) the ILC only being applied to a subsystem instead of being the guiding control architecture.

In this simulation study, we propose the Biologically Inspired Optimal Terminal Iterative Learning Control (BIOTILC) algorithm to control and coordinate the muscles and actuators of the Motor Assisted Hybrid Neuroprosthesis (MAHNP) [28,29]. The MAHNP combines neuromuscular stimulation with exoskeletal bracing that has backdrivable hip and knee joints with Harmonic Drive Transmissions (Harmonic Drive, Peabody, MA). It is an enhanced version of a previous passive-hydraulic hybrid neuroprosthesis [30,31]. BIOTILC is a model-free optimal control method derived from a process described in [32] and applied to exoskeletons to improve control performance over time, requires no prior system identification, and dynamically and simultaneously allocates torque across the muscles crossing several degrees-of-freedom and exoskeletal actuators over each step. The biologically inspired component is based on the ballistic bursting nature of the control of limb dynamics, as the controller only updates burst magnitudes at key points during the gait cycle to achieve specific targets instead of regulating motion throughout the gait cycle. BIOTILC maximizes muscle recruitment, and therefore the physiologic benefits of exercise, with the motors assisting-as-needed to achieve a biologically inspired ballistic swing limb motion.

## 2. Materials and Methods

### 2.1. Device and Simulation Design

The MAHNP modeled here has four motorized joints: two hip joints and two knee joints. Each actuator weighs 2.2 kg (4.85 lbs.), is capable of a peak torque of 36 Nm, and requires less than 6 Nm at all joint speeds (0 to 220°/s) for the limb to overcome its passive resistance and backdrive the joint [28,29]. The actuator is capable of injecting power according to a feedforward model to reduce joint friction by overcoming internal viscous damping, thereby allowing the contracting muscles to drive the system with the brace retaining the ability to assist-as-needed [28]. Solenoid mechanisms (Thomson Linear, Redford, VA, USA) lock all joints during quiet standing or just the knee joint during single limb stance to allow muscles to rest. The exoskeleton records its internal state, including joint kinematics, to then update the controller.

**Figure 1:**
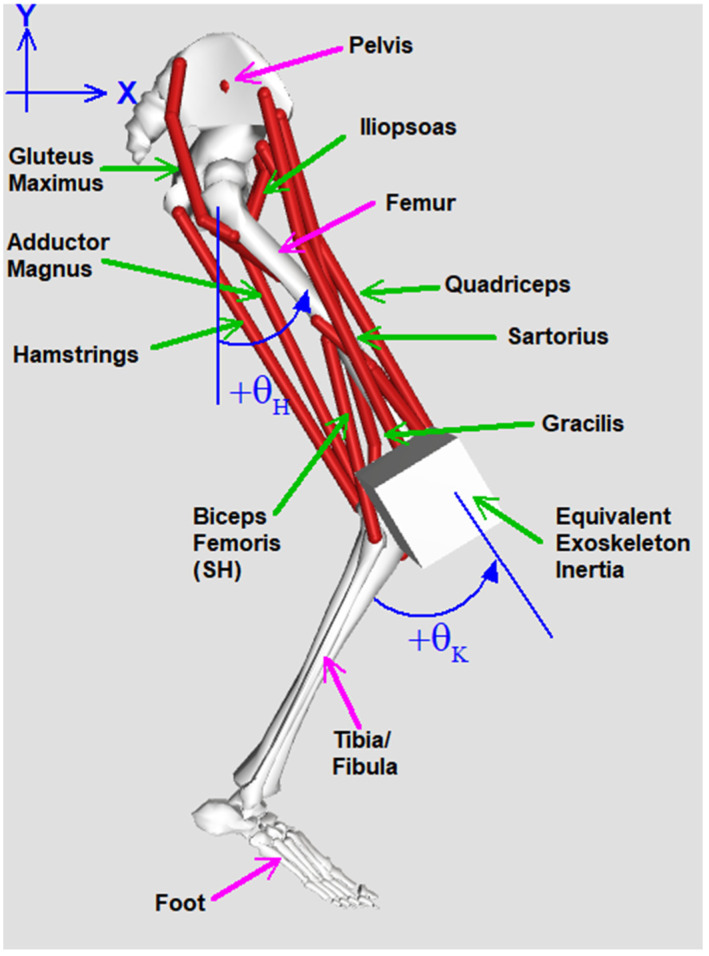
A visualization of the biomechanical model and the relevant axes. Bones represent the modeled mass segments; red lines indicate the modeled muscles; the cube models inertia due to the mass of the actuator. Not shown is the actuator resistance or the actuators themselves.

A simulation of the biological and mechanical subsystems was developed using the OpenSim musculoskeletal modeling software suite (National Center for Simulation in Rehabilitation Research, Stanford, CA, USA). While briefly described above, the exoskeleton model uses the actuator masses, resultant torques, and passive resistances presented in [29]. The simulation is comprised of a single leg in the swing phase connected to a pelvis fixed in space. The biological aspect of the simulation includes an anatomically realistic lower extremity skeleton with height and weight based on a nominal male and contains all relevant muscle groups that are routinely accessible to percutaneous or surface stimulation in our subjects. The physiologic parameters are based on a subject-specific model that reflects the percutaneous electrodes implanted in one of our pilots who has a T4 motor and sensory complete injury. The muscles typically available for stimulation in our system, and for this pilot in particular, are the rectus femoris, vastus lateralis and intermedius, gracilis, sartorius, and tensor fascia latae. The simulation incorporates the relevant masses, inertia, viscous damping, friction compensation, and torque generation characteristics and limitations of the exoskeletal bracing, which are presented in Table 1 and can be found in [29]. a and ϕ implemented here are 0.8 and 4, respectively. These parameters help account for errors, especially near zero speed. The simulated system is illustrated in Figure 1.

### 2.2. Controller Design

To control the pilot’s limbs, the MAHNP activates muscles according to a predefined stimulation pattern consisting of timing onsets and offsets and pulse widths, customized to the individual with SCI on which the model is based. The muscle activation pattern depends on the strength and availability of the stimulated muscles. Executing this sequence of varying stimulation pulse widths recruits the muscles to produce forces on the joints and results in ambulatory motion.

Human gait is often described as “controlled falling”, with gait patterns naturally taking advantage of passive dynamics to walk in an efficient manner. At swing initiation, an impulsive muscle contraction establishes an initial configuration and velocity of the limb. The muscles then relax for the remainder of swing and the leg completes the motion under the influence of momentum and gravity [33,34]. Relevant muscle groups are then activated in terminal swing to prepare for weight acceptance. However, unlike able-bodied ambulation, walking with stimulation only [35] or with commercially available powered exoskeletons does not exhibit this ballistic behavior because of limitations in the strength of the atrophied muscle contractions or the enforced trajectory control by exoskeletal motors. In our case, low passive resistance actuators and powered friction compensation sufficiently reduce the magnitude of viscous damping to allow a short impulse of torque to produce a passive free-swinging motion in the MAHNP under the influence of its own momentum and the force of gravity, much like a two degree-of-freedom pendulum [29]. This characteristic makes the system amenable to mimicking human gait by programming the motors to produce a burst of flexion torque at the hip and knee for a fixed duration at the beginning of the swing phase to augment flexor muscles activated via neural stimulation. Once the hip passes a specified flexion threshold, the MAHNP commands a knee extension burst to help complete the step and ensure stimulation places the limb in the correct position for weight acceptance.

To enhance this biologically inspired burst control with the ability to improve performance over time and iteratively allocate control effort between muscles and motors, we explored an approach that modulates torque bursts and muscle contributions in each step. Conventional ILC is formulated as a repetitive trajectory tracking problem, with control effort applied throughout the motion, providing continuous corrections [19]. In contrast to this, the field of Terminal Iterative Learning Control (TILC) is a formulation of ILC where the main learning objective is controlling the endpoint of the iteration and not maximizing trajectory performance [20]. This formulation is applied to systems where the start, end, and waypoints are specified, and there are no additional constraints on how the system traverses between points [21]. Additionally, it can be applied to systems where it is impossible to record or estimate states that occur between start and end points [36]. The Biologically Inspired Optimal Terminal Iterative Learning Control (BIOTILC) that we develop here does not impose a full trajectory constraint on the MAHNP and only updates the system to enhance the passive portion of swing.

The learning objective of our system is to ensure that the MAHNP achieves an appropriate lower extremity configuration to accept weight at the end of each step. Additionally, knee flexion must reach a sufficient level during swing to ensure floor clearance. Each swing phase is considered an iteration, with the goal of achieving a desired angular configuration described by the vector $y_{d}={\left[y_{hfd},\;y_{kfd},\;y_{ked}\right]}^{T}$ where *y_hfd_* is the desired hip flexion angle, *y_ked_* is the desired knee extension angle at a specified time $t_{f}$, and *y_kfd_* is the desired peak knee flexion during swing phase. The two knee targets represent two different goals during the gait cycle; first the system generates a peak knee flexion for toe clearance; then, the knee must extend by the final time in anticipation of weight bearing. The error for each step is defined by vector ***e_i_***, the deviation of the desired values $y_{d}$ from actual values y at terminal time $t_{f}$ for targets *y_hfd_* and *y_ked_*, and the error for *y_kfd_* compares the desired peak knee angle to the peak value throughout the swing phase.

To achieve this learning objective, the BIOTILC controller updates input vector ***u_i_***. ***u_i_*** is composed of six inputs, $u_{i}={\left[\tau_{hf},\;\tau_{kf},\;\tau_{ke},\;\alpha_{hf},\alpha_{kf},\alpha_{ke}\right]}^{T},$ which are prescribed for each step comprised of motor burst torques (τ) and stimulation scaling factors (α). Stimulation scaling factors modify baseline pulse widths applied via neuromuscular stimulation. The muscles in the pattern corresponding to hip flexion, knee flexion, and knee extension are grouped. The three scaling factors are $\alpha_{hf},\;\alpha_{kf},\;$ and $\alpha_{ke}$, which correspond to the hip flexion, knee flexion, and knee extension pulse width scaling factors respectively. Groupings for biarticular muscles were selected based on the axis of their greatest torque production. Hip extension is not included because it is not necessary to generate the desired swing phase endpoints. The three torque impulses are generated by the actuators, each for a fixed duration; $\tau_{hf}$, $\tau_{kf}$, and $\tau_{ke}$ represent a hip flexion burst at swing initiation, a knee flexion burst at swing initiation, and a knee extension burst that is applied once the hip has passed a programmed angular threshold. BIOTILC modulates the burst amplitudes of the motors and the scaling factors applied to the relevant portion of the stimulation pattern over each step simultaneously.

To account for redundant actuators in the system, the iterative learning control law is formulated as an optimal control problem [37]. Based on Data-Driven Optimal Terminal Iterative Learning Control (DDOTILC), detailed in [32], a terminal cost function is specified that penalizes terminal error at time $t_{f}$ and the rate of change of the input vector over each iteration, with i being the iteration index that represents each subsequent step. BIOTILC adds two novel Least Absolute Shrinkage and Selection Operator (LASSO) terms [38] to the cost function developed for DDOTILC to effectively allocate assistance between motors and stimulation while ensuring the “muscle first” philosophy, the first of which minimizes the control effort of the motors ($\gamma |x_{mot}\cdot u_{i}|)$, and the second of which maximizes recruitment of the muscles ($\frac{\beta }{\left|x_{mus}\cdot u_{i}\right|})$.

(1) $$J\left(u_{i}\right)={\left|\left|e_{i}\right|\right|}^{2}+\lambda {\left|\left|u_{i}-u_{i-1}\right|\right|}^{2}+\gamma \left|x_{mot}\cdot u_{i}\right|+\frac{\beta }{\left|x_{mus}\cdot u_{i}\right|}$$

In Equation (1), $e_{i}=y_{d}-y$ is the terminal error at the end of iteration (step) i. λ is a weighting factor that limits the magnitude of change of inputs, $u_{i}$, between iterations. γ is a weighting factor that governs minimizing the motor contribution, while the β term governs maximizing muscle contribution since the inputs are in the denominator. Stimulation scaling factors α are defined to always be greater than zero to prevent the portion of the equation including β from being undefined. $x_{mot}={\left[1,\;1,\;1,\;0,\;0,\;0\right]}^{T}\;$ and $x_{mus}={\left[0,\;0,\;0,\;1,\;1,\;1\right]}^{T}$ are vectors that extract the motor and muscle components from $u_{i}$.

The optimal control law has an adaptive learning gain that is a function of an estimate of the partial derivatives of the output y with respect to the inputs $u_{i}$ [32]. This gradient estimate is defined as

(2) $$\Delta {\hat{y}}_{i}={\hat{\Psi }}_{i}\Delta u_{i}$$

$\Delta {\hat{y}}_{i}={\hat{y}}_{i}-y_{i-1}$ is the estimate of the change in output relative to change in input $\Delta u_{i}=u_{i}-u_{i-1}$. ${\hat{\Psi }}_{i}\in {\mathbb{R}}^{3\times 6}$ is the online estimate of the gradient, which represents the relationship between the estimated change in outputs relative to change in inputs. The following cost function was defined to develop an update law to minimize the error between the estimated change in outputs $\Delta \hat{y}$ and the actual change in outputs Δy:

(3) $$J\left({\hat{\Psi }}_{i}\right)={\left|\left|\Delta y_{i-1}-{\hat{\Psi }}_{i}\Delta u_{i-1}\right|\right|}^{2}+\mu {\left|\left|{\hat{\Psi }}_{i}-{\hat{\Psi }}_{i-1}\right|\right|}^{2}$$

μ is a weighting term that minimizes the rate of change in the iterative estimate. The iterative gradient update law is computed by taking the partial derivative of the cost function with respect to ${\hat{\Psi }}_{i}$ and setting it to zero. Solving this system for ${\hat{\Psi }}_{i}$ results in the update law [32]:

(4) $${\hat{\Psi }}_{i}={\hat{\Psi }}_{i-1}+\frac{\eta \left(\Delta y_{i-1}-{\hat{\Psi }}_{i-1}\Delta u_{i-1}\right)\Delta u_{i-1}^{T}}{\mu +{\left|\left|\Delta u_{i-1}\right|\right|}^{2}}$$

η is a learning gain that dictates how much the estimate changes due to estimation error over each iteration. The terminal cost function is then rewritten to incorporate the estimate ${\hat{\Psi }}_{i}$:

(5) $$J\left(u_{i}\right)={\left|\left|e_{i-1}-{\hat{\Psi }}_{i}\left(u_{i}-u_{i-1}\right)\right|\right|}^{2}+\lambda {\left|\left|u_{i}-u_{i-1}\right|\right|}^{2}+\gamma \left|x_{mot}\cdot u_{i}\right|+\frac{\beta }{\left|x_{mus}\cdot u_{i}\right|}\;$$

The partial derivative of the cost function is taken with respect to $u_{i}$ and is set to zero. Solving for $u_{i}$ results in the optimal terminal iterative learning control law:

(6) $$u_{i}=u_{i-1}+\frac{\rho \left({\hat{\Psi }}_{i}^{T}e_{i-1}\right)}{{\left|\left|{\hat{\Psi }}_{i}\right|\right|}^{2}+\lambda }-\frac{\gamma x_{mot}sgn\left(x_{mot}\cdot u_{i}\right)}{2\left({\left|\left|{\hat{\Psi }}_{i}\right|\right|}^{2}+\lambda \right)}+\frac{\beta x_{mus}}{x_{mus}\cdot u_{i}\left|x_{mus}\cdot u_{i}\right|}\cdot \frac{1}{2\left({\left|\left|{\hat{\Psi }}_{i}\right|\right|}^{2}+\lambda \right)}\;$$

For the first iteration, an estimate of the gradient ${\hat{\Psi }}_{0}$ is required. In practice, only knowledge of the signs of the elements of ${\hat{\Psi }}_{0}$ are needed. To ensure that this algorithm can be realized in real-time on actual hardware, the noncausal $u_{i}$ terms in the dot products on the right-hand side of the update law are replaced with $u_{i-1}$ when implemented. Finally, a reset algorithm is defined in [32] that resets ${\hat{\Psi }}_{i}$ to ${\hat{\Psi }}_{0}$ if the signs of the elements of ${\hat{\Psi }}_{i}$ no longer match those of the initial estimate. The full derivation of the update control laws is included in Appendix A.

### 2.3. Simulation Implementation

The simulation specified a terminal configuration in terms of a desired hip flexion of $y_{hfd}=30{}^{\circ}$ and a desired knee extension of $y_{ked}=0{}^{\circ}$ at time $t_{f}$ to ensure an appropriate limb orientation to accept weight transfer. To guarantee floor clearance, the desired maximum knee flexion was $y_{kfd}=50{}^{\circ}$ during swing. The duration of the step was $t_{f}=0.5$ s, and the stimulation patterns were time compressed accordingly. The flexion torque bursts commanded to the motors lasted for 0.2 s from the onset of swing, and the knee extension burst, or late swing burst, lasted for 0.2 s. This latter burst was executed once the hip exceeded a specified threshold of 12° of flexion.

The following constants, ρ=0.6, β=0.8, γ=0.5, λ=0.1, μ=1, η=0.2, were applied to BIOTILC. The value of ρ was derived heuristically by first isolating its effect on the terminal error across iterations by setting the β and γ terms to zero. Then, ρ was set to zero and slowly increased, which resulted in an increasing terminal error convergence rate. The system exhibited oscillations in the terminal error once ρ was too large, never reaching a stable, minimal error. At that point, ρ was reduced to the last value that had the highest convergence rate while producing a stable, minimal terminal error. β and γ were then tuned to have tangible effects on maximizing muscle recruitment and minimizing motor control effort, while following a similar tuning routine as described above. An additional stop criterion was when these terms caused a constant offset in error due to the system producing less torque than possible. μ, η, and λ are rate-limiting terms to ensure stable estimates of $\hat{\Psi }$ and $u_{k}$ and thus were set to default values found in [32]. Further tuning of these latter constants may provide a higher convergence rate; however, there is an increased likelihood of poor performance due to incorrect gradient estimates. BIOTILC was initialized with the following gradient estimate:

$${\hat{\Psi }}_{0}=\left[\begin{array}{cc} \begin{array}{ccc} 1 & 0 & 0\\ 0 & 1 & 0\\ 0 & 0 & 1 \end{array} & \begin{array}{ccc} 1 & 0 & 0\\ 0 & -1 & 0\\ 0 & 0 & 1 \end{array} \end{array}\right]$$

This estimate assumes only redundant actuation and no coupling across joints, i.e., it assumes that both the hip motor and the hip flexion muscle stimulation scaling factor influence the amount of terminal hip flexion, whereas the hip flexion torque does not affect the amount of knee flexion. However, if there is coupling between the two terms, such as would result from biarticular muscles, BIOTILC should determine the existence and magnitude of the coupling term and account for it automatically.

The model was run for thirty iterative leg swings to determine if the linear controller could learn to create the desired movement with a non-linear system while only updating six inputs for the entire swing phase. For the first iteration, the motors were commanded zero torque, and all stimulation scaling factors were set to one, meaning that the simulated MAHNP was driven purely by recruited muscle movement via the original stimulation pattern.

To test BIOTILC’s ability to adapt to decreasing force production similar to that observed with muscular fatigue, a simple worst-case mathematical model was applied to the system that decreased the maximum force-generating capacities of all the muscles. For this simulation, the BIOTILC weighting terms were identical to those described above, and the initial inputs $u_{0}$ and gradient estimation ${\hat{\Psi }}_{0}$ were instantiated with the learned values from the 30th iteration of the previous simulations, $u_{30}$ and ${\hat{\Psi }}_{30}$, respectively. For the first five steps of this simulation, maximum muscle force output was decreased by 10% of the initial value. Maximum force output was reduced to 50% strength on the 5th step and remained at that level for all subsequent steps. The model implemented here is not as complex as some models that capture the underlying physiological dynamics of fatigue processes [39], but it represents the relevant features related to declining force production to examine the performance of the controller. Thus, two sets of simulations were performed: One with personalized initial stimulation patterns to muscles with nominal strength and initial motor torques set to zero, and the other with an arbitrary rate of decline of stimulated muscle force producing capacity with initial motor torques set to the values determined at completion of the simulations with no decrease in muscle strength.

## 3. Results

**Figure 2:**
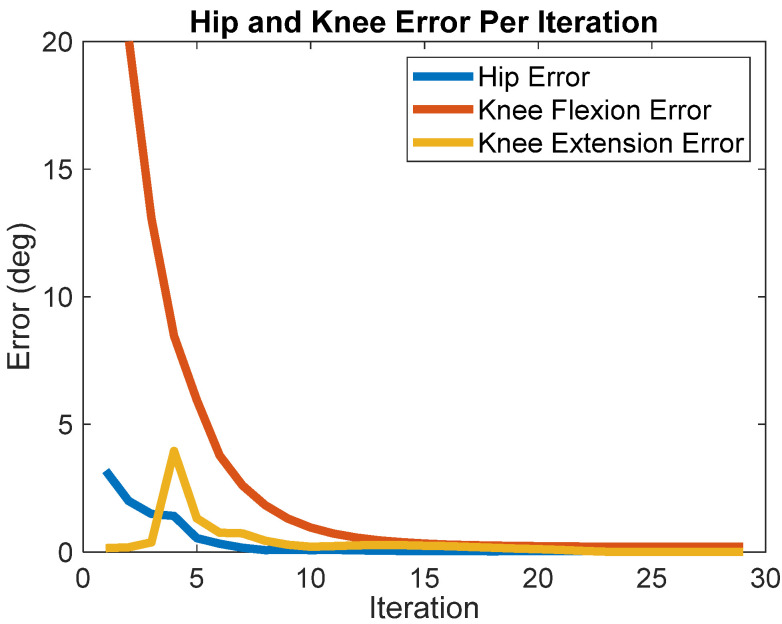
Absolute terminal error over each iteration (swing phase) for hip and knee flexion and knee extension.

**Figure 3:**
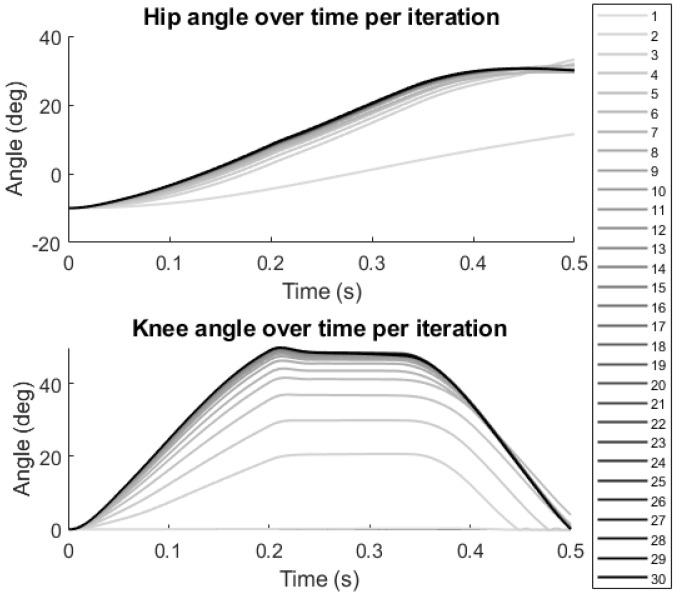
Joint angle progression as the controller updates inputs with each successive swing phase. The line becomes darker to indicate progressive iterations.

BIOTILC achieved the learning objective during the course of thirty iterations for the swing phase with errors shown in Figure 2 and the progression of hip and knee angle trajectories displayed in Figure 3. Visualizations of the swing trajectory progressions are included in the supplementary materials without and with the force reductions, (Video S1) and (Video S2) respectively. By the 15th iteration, the system adapted by maximizing muscle recruitment and assisting as needed with motor bursts to minimize error. BIOTILC was capable of both ensuring foot clearance as well as attaining the terminal stance configuration required to accept weight.

After 30 iterations, the estimate of $\hat{\Psi }$ was

$${\hat{\Psi }}_{30}=\left[\begin{array}{cc} \begin{array}{ccc} 1.09 & -0.02 & -0.07\\ -0.07 & 1.69 & -0.02\\ -0.01 & 0.07 & 1.05 \end{array} & \begin{array}{ccc} 1.67 & 0.60 & 0.52\\ 0 & -1.03 & 0.04\\ -0.05 & -0.05 & 1.01 \end{array} \end{array}\right]$$

The algorithm determined that the most significant joint coupling terms were in row 1, columns 5 and 6, of 0.6 and 0.52, respectively. These terms indicate that change in terminal hip flexion is affected not just by the hip motor (1.09) and hip flexion stimulation scaling factor (1.67), but by an increase in the stimulation scaling factors governing knee flexion (0.60) and knee extension (0.52) as well, demonstrating that the system could learn the contributions of biarticular muscles as the update gradient iterated. Finally, there were small, almost negligible estimated coupling influences for the rest of the terms.

**Figure 4:**
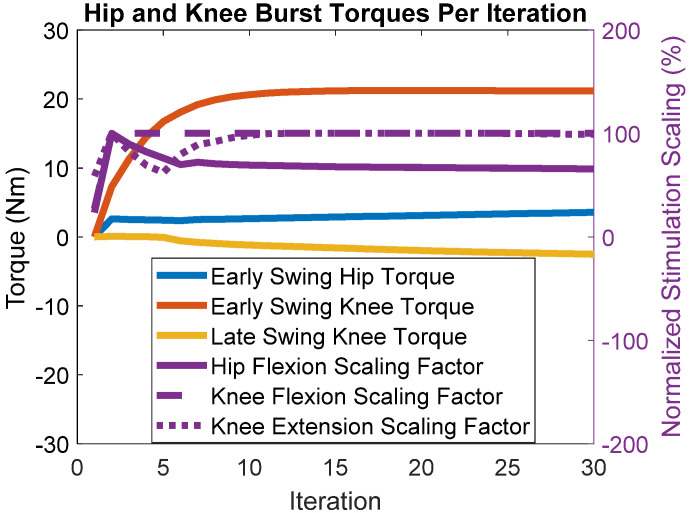
Muscle and motor recruitment over each iteration. For the left-hand axis, positive values are flexion, negative is extension. Early swing torques are executed at the onset of swing. Late swing knee torque extends the knee to prepare for weight acceptance. Purple lines represent neural stimulation scaling factors and are normalized using the scale on the right-hand axis. A scaling factor of 100% means that the muscles reached the maximum, i.e., the scaling factor produces a maximal pattern with peak pulse.

The simulations show that we achieved the “muscle-first” objective of maximizing muscle recruitment and minimizing motor control effort, seen in Figure 4. The figure shows updated commanded motor torques and stimulation scaling as they adjust with each iteration (step) based on the errors. Commanded motor torques are indicated by the orange, blue, and yellow lines paired with the left axis. The right axis is paired with the purple lines, which represent normalized stimulation scaling factors. A scaling of 100% means that the multiplicative scaling factor applied to the pattern resulted in the pattern having a peak pulse-width of 255 microseconds, which is the maximum the stimulator control board can output. The stimulation scaling factor for knee flexion was maximized within the first five iterations, and the knee extension scaling factor reached the maximum within 15 iterations. The hip flexion scaling factor was maximized within five iterations, but in further steps, BIOTILC determined that with the hip motor and hip flexors such as the sartorius and tensor fascia latae, gracilis, and rectus femoris acting on the joint, it was unnecessary to maximize hip flexor muscle recruitment to achieve the learning objective. The motors made up for any deficits, with 21.1 Nm of flexion torque required from the knee motor to achieve foot clearance. To achieve the terminal configuration, only 3.6 Nm hip flexion torque and 2.5 Nm knee extension torque were required of the motors. None of the motors reached the maximal peak torque of 36 Nm, while the stimulation scaling factors aside from the hip scaling factor were near maximal.

**Figure 5:**
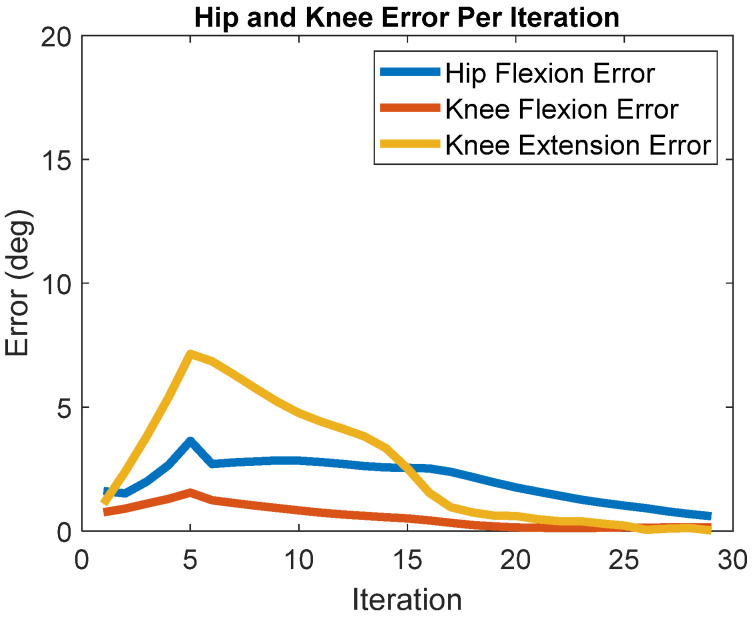
Absolute terminal error over each iteration in the presence of simulated fatigue.

Introducing a reduction in stimulated muscle force output (i.e., “fatigue”) at the end of the initial set of swing motions gradually increased joint angle errors, but the system adapted and eventually returned to similar errors as those generated prior to the decrease in the muscle force-generating capacity. Figure 5 depicts the absolute terminal error over each iteration. In the first five steps, the terminal error increased as the muscles weakened. The terminal error peaked at 3.6°, 1.6°,  and 7° for the hip flexion, knee flexion, and knee extension errors, respectively. Once the system experienced constant fatigue, BIOTILC adapted, and by the 30th iteration, there was less than 0.6° of error for each desired terminal configuration.

**Figure 6:**
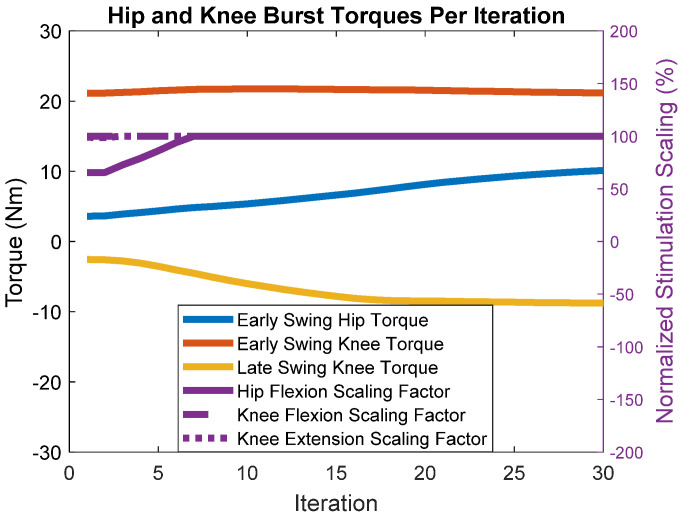
Muscle and motor recruitment over each iteration in the presence of simulated fatigue. The initial values of muscle and motor recruitment are based on the 30th iteration of the non-fatigue simulation.

Figure 6 shows the stimulation and commanded motor torque inputs at each step after the introduction of the gradually decreasing muscle torques. The lines indicate how control effort is distributed across muscular recruitment and motor burst torques over each iteration. Exhibiting the “muscle-first philosophy”, the rate of increase in hip flexion muscular recruitment is faster than the increase in motorized burst torque, becoming maximized at the 7th iteration. Motorized knee flexion torque stayed largely the same, with the motorized hip flexion and knee extension torques reaching 10.1 Nm and 8.8 Nm, respectively, in the 30th iteration. These simulations illustrate BIOTILC’s ability to adapt to the presence of fatigue in the system.

## 4. Discussion

The simulation study presented here demonstrates that a model-free control approach that prioritizes muscle activation over motor power has potential for controlling hybrid exoskeletal walking assist devices. The implementation controls a few inputs that regulate motor torque bursts at key points in the swing phase, and parameters that scale stimulus inputs for a set of muscles can mimic the ballistic bursting control of limb dynamics generated by the human body, effectively generating a swing phase motion without regulating control and enforcing a fixed trajectory throughout the entire swing phase. These results suggest this approach could be implemented as a way to optimize motor assistance and stimulation inputs via simulation prior to clinical implementation with a person in the loop. The findings also indicate the feasibility of eventually updating control in real-time when eventually implemented with a physical exoskeleton and user with SCI to balance the contributions of motorized assistance and stimulated muscle outputs for ballistic control of swing.

The innovation of the work shown here is the application of terminal iterative learning control to the new application of generating motions necessary for the gait cycle by balancing contributions of external motors and internally generated forces of stimulation-induced contractions of the paralyzed muscles. The work builds on existing learning controllers and applies them in a manner that implements simultaneous stimulation and motor assistance while prioritizing activation of the user’s muscles. Furthermore, ballistic motions are produced without following or enforcing prescribed trajectory, but instead only specifying three targets (toe off, midswing foot-floor clearance, and terminal foot-floor contact) during the swing phase. The controller only updates six inputs once during the entire swing phase to achieve those three targets, contributing to the simplicity of implementation, reducing computational burden, and enhancing opportunities for clinical implementation. Instead of controlling individual muscles, the controller treats groups of muscles as a single actuator, further simplifying the necessary processing. The results demonstrate that these processes are relevant to the field of hybrid exoskeletons incorporating powered motors and neural stimulation with potential for clinical applications.

Direct comparison with prior implementations of hybrid exoskeletons that combine neural stimulation with external motors is difficult due to the variety of outcome measures and activities reported in the literature [14,40,41,42]; however, some generalizations can be made. Similar to other implementations, the system effectively learned over time, reduced errors [27], and adapted to simulated fatigue represented by a gross decline in muscle force-generating capacities [26]. Another simulation study generated similar swing phase timings of around 0.5 s [16], which are necessary to enable the gait speeds required for community ambulation, whereas all physical implementations reported slower gait speeds or did not report walking speed at all [7,24,25].

One of the muscles recruited for knee flexion was the gracilis, and for knee extension, the rectus femoris was activated. Both muscles are biarticular, affecting movement simultaneously across the hip and knee joints. The gracilis contributes to both knee flexion and hip flexion, while the rectus femoris affects both hip flexion and knee extension. It is clear that BIOTILC estimated the coupling terms that indicate the influence of both of these muscles on hip flexion. The entry in row 2, column 2 of ${\hat{\Psi }}_{30}$ indicates that the knee flexion motor burst had a large influence on achieving the desired angle for floor clearance. This correlates with the knee flexion burst being the highest commanded torque of all motorized bursts. These results suggest the controller can learn both the mechanical interactions as well as the complex contributions of the various stimulated muscles.

A focus on single leg swing allows the controller to be evaluated without the impact of varied initial conditions due to changing steps. Important next steps will be to evaluate the controller during continuous multi-step walking in simulation and in people with SCI. These assessments will require adding a controller for the stance phase of gait. Now that it has been demonstrated during single limb swing, it will be important to evaluate continuous multi-step walking. Further implementation will also require separate controller inputs and psi matrices for each leg. Since the stimulation response, muscle conditioning, and muscle fatigue differ between limbs, the stimulation and motor inputs will need to be updated independently for each leg. Additionally, the ILC approach could be combined with guided reinforcement learning control to improve the efficiency of the initial search while becoming more robust to variations or perturbations [43].

A limitation of the control approach is that it does not ensure reaching the targets with every step. The approach could be implemented in simulation initially or with adequate safety supports such as overhead support harnesses during online learning when implemented clinically with a human in the loop. Additionally, closed-loop feedback could be added for the motors to ensure meeting necessary safety targets. Nevertheless, the present implementation in simulation enables ascertaining the ideal performance that could be expected from the learning controller alone.

In the ideal case, the constants derived for this simulation can be transferred to the physical system with little to no changes or further tuning. However, some tuning will be required since the simulation cannot capture all the subtle dynamics of the physical system. Another limitation is that in implementation, the signs of the initial gradient estimate are required, and some of the weighting factors may require retuning to ensure convergence and prevent oscillation about the terminal configuration over each iteration.

## 5. Conclusions

BIOTILC is a cooperative iterative learning controller designed to control a “muscle-first” motor-assisted hybrid neuroprosthesis that combines neuromuscular stimulation and motorized actuation. It is a model-free algorithm capable of iteratively improving performance by maximizing the recruitment of the muscles and minimizing the motors simultaneously over each step. Simulations of the MAHNP in swing phase show the efficacy of this algorithm in achieving the correct terminal swing configuration, ensuring foot clearance, and exemplifying the “muscle-first” paradigm, as well as adapting to muscular fatigue.

Future avenues for investigation include iteratively learning the stimulation patterns for each muscle over time, as opposed to scaling a pre-existing pattern. Additionally, the algorithm can be extended to control the single stance limb as well as swing limb to drive the system forward. The simulation itself can be enhanced by incorporating the effect of heel strike, as well as removing the fixed constraint on the pelvis and allowing it to move through space to represent natural forward progression of the body.

It has been suggested that an exoskeletal assist-as-needed paradigm requires a forgetting factor applied to the motorized portion of the system to keep the pilot challenged [44]. Thus, BIOTILC could be reformulated with a forgetting factor based on an extension of DDOTILC [45]. Additionally, it would be possible to extend the optimal control algorithm with higher-order learning terms [46], control of multiple intermediate pass points [47], and initial value dynamic compensation [48]. Finally, efforts are underway to implement BIOTILC on the MAHNP and test the algorithm with SCI pilots.

## Figures and Tables

**Table 1:** Simulated MAHNP Characteristics.

Characteristic	Quantity	Description
Actuator Masses	2.2 kg	
Actuator Torque Limits	±36 Nm	Peak torque limit
Viscous Damping Model	$\sigma \left(\omega \right)\cdot \left|b\omega +g\right|$	Results in <6 Nm of torque required to backdrive actuator at joint speeds of ω=0–220°/s [28]. $\sigma \left(\omega \right)=2/\left(1+e-\omega \right)-1$, is a scaled and shifted sigmoid to center the torque at zero. b and g represent the polynomial fit terms for the friction model for each actuator.
Feedforward Friction Compensation	$a\cdot \sigma \left(\frac{\omega }{\phi }\right)\cdot \left|b\omega +g\right|$	Compensator derived in [28]. a and ϕ are tuning parameters to account for errors in the polynomial fit and reduce sensitivity to noise when operating near zero speed.
Actuator Electrical Current Dynamics	$\tau \frac{dc}{dt}+c=s\left(t\right)$	c is the current, $s\left(t\right)$ is the current setpoint, τ=0.0025 resulting in a current rise time of 10 ms.

## Data Availability

The data presented in this study are available on request from the corresponding author.

## Supplementary Materials

The following supporting information can be downloaded at: https://www.mdpi.com/article/10.3390/bioengineering9020071/s1, Video S1: Kinematic progressions during learning, Video S2: Kinematic progressions during learning with force reductions.

## Appendix A

### Appendix A.1. Controller Derivation

#### Appendix A.1.1. Variables

$u_{i}$: the control input vector for each iteration i.

$$u_{i}=\left[\begin{array}{c} \tau_{mot}\\ \alpha_{mus} \end{array}\right]=\left[\begin{array}{c} \tau_{hf}\\ \tau_{kf}\\ \underline{\tau_{ke}}\\ \alpha_{hf}\\ \alpha_{kf}\\ \alpha_{ke} \end{array}\right]$$

$\tau_{hf},\tau_{kf},\;\tau_{ke}$: the input motor torques, in Nm, for hip flexion $\left(\tau_{hf}\right)$, knee flexion $\left(\tau_{kf}\right)$, and knee extension $\left(\tau_{ke}\right),$ respectively

$\alpha_{hf},\alpha_{kf},\;\alpha_{ke}$: the input scaling factors for electrical stimulation pulse width for hip flexion $\left(\alpha_{hf}\right)$, knee flexion $\left(\alpha_{kf}\right)$, and knee extension $\left(\alpha_{ke}\right)$.

$x_{mot},\;x_{mus}$: vectors used to extract the motor and electrical stimulation components from the input vector $u_{i}$.

$$x_{mot}=\left[\begin{array}{c} 1\\ 1\\ 1\\ 0\\ 0\\ 0 \end{array}\right];x_{mus}=\left[\begin{array}{c} 0\\ 0\\ 0\\ 1\\ 1\\ 1 \end{array}\right]$$

$y_{i}$: the output vector of angles, in degrees, for each iteration i for hip flexion $\left(y_{hf}\right)$, knee flexion $\left(y_{kf}\right)$, and knee extension $\left(y_{ke}\right)$.

$$y_{i}=\left[\begin{array}{c} y_{hf}\\ y_{kf}\\ y_{ke} \end{array}\right]$$

${\hat{y}}_{i}$: the estimated output vector for each iteration i.

$${\hat{y}}_{i}=\left[\begin{array}{c} {\hat{y}}_{hf}\\ {\hat{y}}_{kf}\\ {\hat{y}}_{ke} \end{array}\right]$$

$y_{d}$: the desired output vector.

$$y_{d}=\left[\begin{array}{c} y_{hf,d}\\ y_{kf,d}\\ y_{ke,d} \end{array}\right]$$

$e_{i}$: the terminal tracking error.

$$e_{i}=y_{d}-y_{i}$$

Ψ: a 3 × 6 gradient matrix that links the inputs $\left(u_{i}\right)$ to the outputs $\left(y_{i}\right)$.

${\hat{\Psi }}_{i}$: the estimated gradient matrix, which is updated each iteration.

#### Appendix A.1.2. Parameters

λ=0.1 is a weighting factor for minimizing the change in inputs.

γ=0.5 is a weighting factor for minimizing the motor inputs.

β=0.8 is a weighting factor for maximizing the muscle stimulation inputs.

ρ=0.6 is a positive real scalar that determines the step size.

μ=1 is a weighting factor for minimizing the change of the estimated gradient.

η=0.2 is a positive real scalar that determines the step size.

#### Appendix A.1.3. Derivation

Our controller design is based on [32].

(A1) $$y_{i}=y_{i-1}+\Psi_{i}(u_{i}-u_{i-1})=y_{i-1}+\Psi_{i}\Delta u_{i}$$

is the linear incremental relationship between system output and control input and provides the update of joint angles at current step from the values at the previous step. The relationship is based on a simple Taylor’s series expansion. $y_{i}$ is the measured output, $u_{i}$ is the control input, and $\Psi_{i}$ is the gradient that maps the inputs to the outputs. Since $\Psi_{i}$ is unknown, we use an iterative estimate of the gradient, ${\hat{\Psi }}_{i}$, producing

(A2) $${\hat{y}}_{i}=y_{i-1}+{\hat{\Psi }}_{i}\Delta u_{i}$$

where ${\hat{y}}_{i}$ is the estimated output. If $y_{d}$ is our desired terminal output, we define our terminal tracking error as

(A3) $$e_{i}=y_{d}-y_{i}$$

For the controller, we require an update law for our inputs $(u_{i}$) and for the estimation of our gradient matrix $\left({\hat{\Psi }}_{i}\right)$. First, we begin by defining a cost function, $J\left(u_{i}\right)$, to minimize terminal tracking error, minimize change in inputs, minimize motor input, and maximize electrical stimulation input.

(A4) $$J\left(u_{i}\right)={\left|\left|ei\right|\right|}^{2}+\lambda {\left|\left|u_{i}-u_{i-1}\right|\right|}^{2}+\gamma \left|x_{mot}\;\cdot u_{i}\right|+\beta \frac{1}{\left|x_{mus}\cdot u_{i}\right|}$$

where λ, γ, and β are weighting terms, and $x_{mot}$ and $x_{mus}$ are vectors used to extract the motor and muscle stimulation components from the inputs, respectively.

Using Equations (A1) and (A3), (A5) can be rewritten as

(A5) $$J\left(u_{i}\right)={\left|\left|e_{i-1}-\Psi_{i}\left(u_{i}-u_{i-1}\right)\right|\right|}^{2}+\lambda {\left|\left|u_{i}-u_{i-1}\right|\right|}^{2}+\gamma \left|x_{mot}\;\cdot u_{i}\right|+\beta \frac{1}{\left|x_{mus}\cdot u_{i}\right|}$$

Taking the partial derivative of Equation (4) with respect to $u_{i}$ and setting the result to zero results in

(A6) $$\frac{\partial J\left(u_{i}\right)}{\partial u_{i}}=0$$

(A7) $$-2\Psi_{i}^{T}e_{i-1}+2u_{i}\left({\left|\left|\Psi_{i}\right|\right|}^{2}+\lambda \right)-2u_{i-1}\left({\left|\left|\Psi_{i}\right|\right|}^{2}+\lambda \right)+\gamma x_{mot}sgn\left(x_{mot}\cdot u_{i}\right)-\beta \frac{x_{mus}}{x_{mus}\cdot u_{i}\left|x_{mus}\cdot u_{i}\right|}=0$$

Then, solving for $u_{i}$, using the matrix inverse lemma from [49] to get the ${\left|\left|\Psi_{i}\right|\right|}^{2}+\lambda$ term in the denominator results in an optimal update law for the control inputs

(A8) $$u_{i}=u_{i-1}+\rho \frac{\Psi_{i}^{T}e_{i-1}}{{\left|\left|\Psi_{i}\right|\right|}^{2}+\lambda }-\gamma \frac{x_{mot}sgn\left(x_{mot}\cdot u_{i}\right)}{2\left({\left|\left|\Psi_{i}\right|\right|}^{2}+\lambda \right)}+\beta \frac{x_{mus}}{x_{mus}\cdot u_{i}\left|x_{mus}\cdot u_{i}\right|}\cdot \frac{1}{2\left({\left|\left|\Psi_{i}\right|\right|}^{2}+\lambda \right)}$$

where ρ is a positive real scalar that determines the step size.

To ensure that this algorithm can be realized in real-time on actual hardware, the noncausal $u_{k}$ terms in the dot products on the right-hand side of the update law are replaced with $u_{k-1}$ when implemented. Further, since $\Psi_{i}$ is unknown for the control input update law, Equation (A8), by using the estimated gradient ${\hat{\Psi }}_{i}$, the control law becomes

(A9) $$u_{i}=u_{i-1}+\rho \frac{{\hat{\Psi }}_{i}^{T}e_{i-1}}{{\left|\left|\Psi_{i}\right|\right|}^{2}+\lambda }-\gamma \frac{x_{mot}\mathrm{sgn}\left(x_{mot}\cdot u_{i}\right)}{2\left({\left|\left|\Psi_{i}\right|\right|}^{2}+\lambda \right)}+\beta \frac{x_{mus}}{x_{mus}\cdot u_{i-1}\left|x_{mus}\cdot u_{i-1}\right|}\cdot \frac{1}{2\left({\left|\left|\Psi_{i}\right|\right|}^{2}+\lambda \right)}$$

Similar steps are taken for the estimated gradient matrix $\left({\hat{\Psi }}_{i}\right)$ update law. This time, we define a cost function, $J\left({\hat{\Psi }}_{i}\right)$, to minimize the error between our measured and estimated outputs and minimize the change in the estimated gradient.

(A10) $$J\left({\hat{\Psi }}_{i}\right)={\left|\left|\Delta y_{i-1}-{\hat{\Psi }}_{i}\Delta u_{i-1}\right|\right|}^{2}+\mu {\left|\left|{\hat{\Psi }}_{i}-{\hat{\Psi }}_{i-1}\right|\right|}^{2}$$

where μ is a weighting or regularization parameter.

Taking the partial derivative of Equation (A10) with respect to ${\hat{\Psi }}_{i}$, setting the result to zero, and solving for ${\hat{\Psi }}_{i}$ results in an optimal update law for estimating the gradient.

(A11) $${\hat{\Psi }}_{i}={\hat{\Psi }}_{i-1}+\eta \frac{\left(\Delta y_{i-1}-{\hat{\Psi }}_{i-1}\Delta u_{i-1}\right)\Delta u_{i-1}^{T}}{\mu +\Delta {\left|\left|u_{i-1}\right|\right|}^{2}}$$

where η is a positive real scalar that determines the step size.
